# Genome-Wide SNPs Clarify a Complex Radiation and Support Recognition of an Additional Cat Species

**DOI:** 10.1093/molbev/msab222

**Published:** 2021-07-28

**Authors:** Fernanda J Trindade, Maíra R Rodrigues, Henrique V Figueiró, Gang Li, William J Murphy, Eduardo Eizirik

**Affiliations:** 1 PUCRS, Escola de Ciências da Saúde e da Vida, Laboratório de Biologia Genômica e Molecular, Porto Alegre, RS, Brazil; 2 USP, Department of Genetics and Evolutionary Biology, Institute of Biosciences, São Paulo, Brazil; 3 Center for Species Survival, Smithsonian Conservation Biology Institute, National Zoological Park, Front Royal, VA, USA; 4 Department of Veterinary Integrative Biosciences, Texas A&M University, College Station, TX, USA; 5 College of Life Sciences, Shaanxi Normal University, Xi’an, Shaanxi, China; 6 Instituto Pró-Carnívoros, Atibaia, SP, Brazil

**Keywords:** phylogeny, neotropics, genomics, speciation, mammalia

## Abstract

Phylogenetic reconstruction and species delimitation are often challenging in the case of recent evolutionary radiations, especially when postspeciation gene flow is present. *Leopardus* is a Neotropical cat genus that has a long history of recalcitrant taxonomic problems, along with both ancient and current episodes of interspecies admixture. Here, we employ genome-wide SNP data from all presently recognized *Leopardus* species, including several individuals from the tigrina complex (representing *Leopardus guttulus* and two distinct populations of *Leopardus tigrinus*), to investigate the evolutionary history of this genus. Our results reveal that the tigrina complex is paraphyletic, containing at least three distinct species. While one can be assigned to *L. guttulus*, the other two remain uncertain regarding their taxonomic assignment. Our findings indicate that the “tigrina” morphology may be plesiomorphic within this group, which has led to a longstanding taxonomic trend of lumping these poorly known felids into a single species.

Genome-wide data hold great potential to address complex evolutionary problems, such as resolving the phylogenetic relationships and dissecting introgression histories among closely related species (e.g., Li et al. 2016, 2019; Edelman et al. 2019; Pulido-Santacruz et al. 2020). Within the mammalian order Carnivora, several genera have undergone recent radiations, leading to complex networks that challenge phylogenetic resolution using traditional approaches (e.g., Figueiró et al. 2017). In the Neotropics (encompassing South and Central America, Mexico, and Southern USA), at least two genera (*Leopardus* in the Felidae and *Lycalopex* in the Canidae) have diversified recently, each of them following a single episode of colonization from North America during the Pliocene or Pleistocene (Eizirik 2012). Accurately resolving the phylogenetic structure of these clades is critical to stabilize their taxonomy, enable adequate conservation assessment and actions on behalf of these threatened organisms, and allow a better understanding of the intricate evolutionary and biogeographic history of Neotropical biotas.

*Leopardus* is a Neotropical-endemic clade of small to medium-sized wild cats that diverged from other felid lineages *ca.* 10 million years ago (MYA), and underwent a radiation starting *ca.* 3–4 MYA (Li et al. 2016). It comprises at least seven extant species that have been traditionally recognized by taxonomists since the mid-20th century: ocelot (*Leopardus pardalis*), margay (*Leopardus wiedii*), Andean mountain cat (*Leopardus jacobita*), pampas cat (*Leopardus colocola*), Geoffroy’s cat (*Leopardus geoffroyi*), huiña (*Leopardus guigna*), and tigrina (*Leopardus tigrinus*). The latter has recently been found to represent at least two distinct species, the northern tigrina (*L. tigrinus*) and southern tigrina (*Leopardus guttulus*), based on the analysis of multiple molecular markers (Trigo et al. 2013). Subsequent morphological analyses supported the distinctiveness of *Leopardus guttulus*, and further proposed the separation of *Leopardus emiliae* (occurring in northeastern Brazil) from *L. tigrinus*, which would be restricted to northern and western South America, as well as Central America (Nascimento and Feijó 2017) (see species distribution in supplementary fig. S1, Supplementary Material online). This arrangement has so far not been tested with molecular data, for the reasons outlined below.

Several molecular studies focusing on this genus have revealed that it has had a complex evolutionary history, including different episodes of interspecies hybridization. Previous work has shown that the southern tigrina (*L. guttulus*) is currently hybridizing with Geoffroy’s cat in southern Brazil (Trigo et al. 2008, 2013, 2014). In contrast, tigrina populations from northeastern Brazil (NE tigrina), identified as *L. tigrinus* or *L. emiliae* depending on the assumed classification, bear molecular signatures of ancient hybridization with pampas cats (Trigo et al. 2013). This ancient interspecies admixture has resulted in remarkable cytonuclear discordance in the NE tigrina, with complete replacement of its mitochondrial genome with introgressed mtDNA from the pampas cat (Trigo et al. 2013; Santos et al. 2018). The latter taxon (*L. colocola*) has recently been proposed to actually comprise five distinct species (Nascimento et al. 2020); under this scheme, the hybridization of NE tigrina would have occurred with the central/northeastern Brazilian pampas cat (*Leopardus braccatus*), based on mtDNA phylogeographic analyses (Santos et al. 2018). Importantly, this mitochondrial replacement has precluded any mtDNA-based phylogenetic analysis comparing NE tigrinas with other members of the tigrina complex. At the same time, the nuclear markers analyzed so far (Trigo et al. 2013) did not contain enough phylogenetic signal to reliably resolve their relationships, suggesting that NE tigrinas and *L. guttulus* could be sister-species (based on Y-chromosome markers) or that the former was in fact more closely related to Geoffroy’s cat (based on X-chromosome markers).

Furthermore, these earlier studies did not include representatives of additional, geographically distant, tigrina populations. This is especially relevant since early mtDNA data (Johnson et al. 1999; Trigo et al. 2008) had indicated that Central American tigrinas (presently recognized as *Leopardus tiginus oncilla* [Kitchener et al. 2017]) were very divergent from southern South American populations (now recognized as *L. guttulus*). Genome-wide SNP data supported this deep divergence (Li et al. 2016), but that study included only the Central American lineage and the NE tigrina, with no representation of *L. guttulus*. Therefore, no phylogenetic assessment thus far has included all three tigrina units.

Here, we expand on the genome-wide SNP data set reported by Li et al. (2016) by including several *L. guttulus* individuals as well as additional Geoffroy’s cat specimens. We also genotyped previously identified hybrids between these two species, as well as a captive-bred hybrid between *L. guttulus* and the pampas cat, aiming to assess the effects of including admixed individuals in genome-wide assessments of species-level monophyly and phylogenetic relationships. Our results indicate that this impact can be quite relevant in phylogenetic analyses of recent radiations, and robustly demonstrate that the tigrina complex comprises at least three different species.

## Results and Discussion

**Fig. 1:**
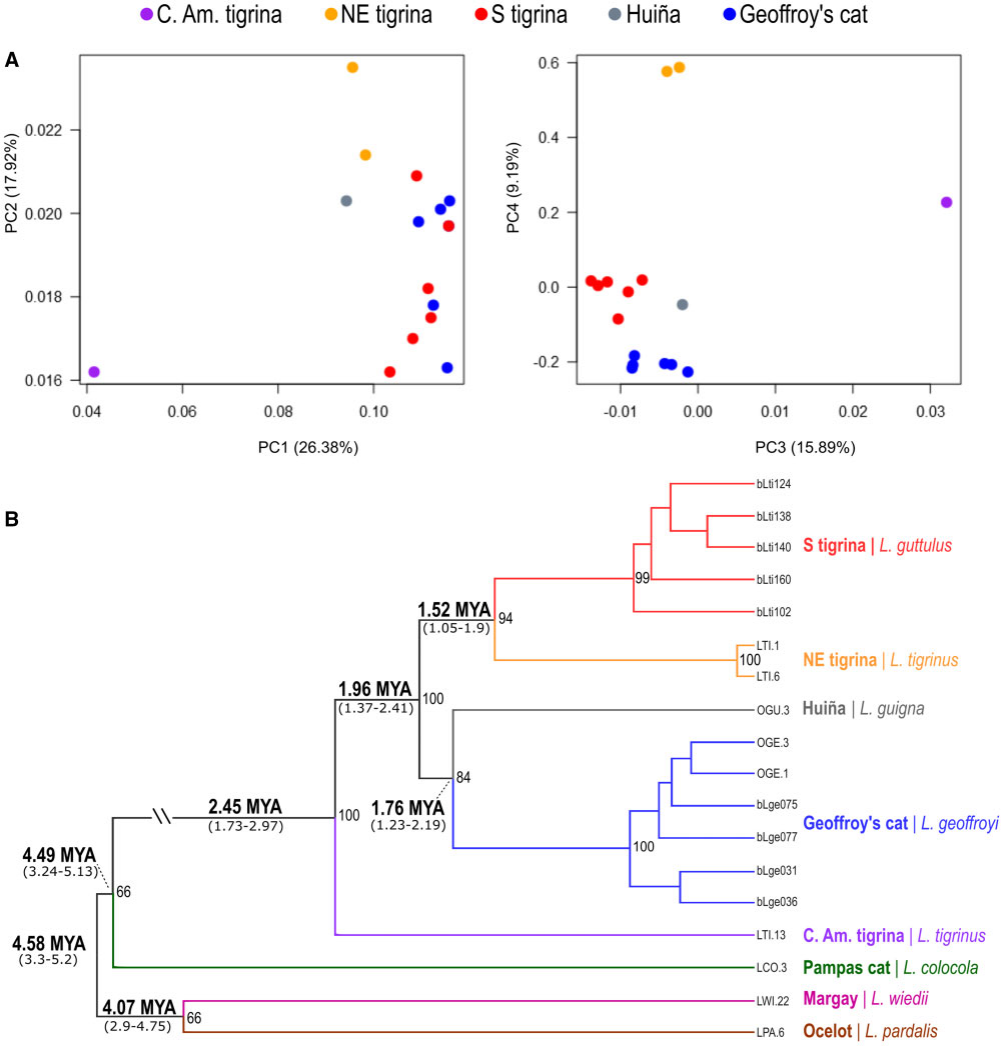
Population genetic and phylogenetic analyses of *Leopardus* based on genome-wide SNP data (see text and Supplementary Information, Supplementary Material online for details). (*A*) Principal component analysis for the focal taxonomic group, comprising the tigrina complex, Geoffroy’s cat and huiña; PCs 1–4 are shown, along with their respective variance explanatory power. (*B*) Maximum likelihood phylogeny of *Leopardus* based on a supermatrix comprising 60,931 SNPs (including 4,708 variable sites); hybrid individuals and those with extensive missing data were excluded (see Supplementary Information, Supplementary Material online for details). Bootstrap support values are shown next to nodes (nodes with no values indicate support below 60%). Numbers above branches are indicate divergence times (in Million years ago) for the adjacent node, with credibility intervals shown below the respective branch (see supplementary fig. S11, Supplementary Material online for true branch heights).

We performed multiple sets of analyses to investigate the impacts of varying taxon sampling, filtering schemes for missing data, treatment of heterozygous sites, and inclusion of hybrid individuals (see Materials and Methods and Supplementary Information, Supplementary Material online for details). Principal component analyses (PCAs) revealed a clear separation among the recognized species and indicated that the three sampled tigrina units (southern (S) tigrina [*L. guttulus*], NE tigrina, and Central American tigrina) were very distinct from each other (fig. 1*A*, supplementary fig. S2, Supplementary Material online). This finding was corroborated by the Admixture analyses, regardless of the taxon sampling scheme (supplementary figs. S3–S5, Supplementary Material online). In the PCA plots, S tigrina and NE tigrina were at least as distinct from each other as Geoffroy’s cats *versus* huiñas. In the Admixture plots, S tigrina and NE tigrina exhibited completely different ancestry assignments from *K* = 3 (taxon subgroups 2 and 3) or *K* = 4 (subgroup 1) upwards, much below the optimal K inferred for each data set. In addition to the separation between the two South American tigrina units, the PCA and Admixture results also supported the distinctiveness of the Central American tigrina (e.g., it was the most distinct unit in PC1 for the focal taxonomic group).

In addition to assessing the distinctiveness among the three tigrina units, we also investigated interspecies hybridization. Our data supported the inference of admixed ancestry in one field-collected individual (bLti135) that had been previously reported to be a hybrid between S tigrina and Geoffroy’s cat using traditional molecular markers (Trigo et al. 2013). We also found that another individual (LCO.2), previously suspected to derive from admixture in captivity between S tigrina and pampas cat (Trigo et al. 2008), was indeed a hybrid (likely F1) between these species. Importantly, we did not find any evidence of nuclear introgression from pampas cat into NE tigrina (supplementary fig. S3, Supplementary Material online), in striking contrast to the complete substitution of the latter’s mtDNA with that of the former (Trigo et al. 2013; Santos et al. 2018). This indicates that signatures from the ancient hybridization episode between these species may have been erased from the nuclear genome by cumulative backcrossing, and highlights the remarkable cytonuclear discordance present in NE tigrinas.

We performed extensive phylogenetic analyses with our SNP data set using several combinations of individuals, filters for missing data, and treatments of heterozygous sites (see Supplementary Information, Supplementary Material online). We also employed different phylogenetic approaches: maximum likelihood (ML) on a supermatrix comprising the concatenation of all sites (including both variable and invariant positions), and SNP-based phylogenetic reconstructions. All analyses converged on the conclusion that the tigrina complex is paraphyletic (fig. 1*B*;supplementary fig. S6–S10, Supplementary Material online). ML trees reconstructed the NE tigrina and S tigrina as sister-groups, but strongly supported the placement of the Central American tigrina at a more external position, outside of the clade that also included two other, well-recognized *Leopardus* species (Geoffroy’s cat and huiña). Coalescent-based trees also strongly supported this inference, and further indicated paraphyly of NE tigrinas and S tigrinas relative to Geoffroy’s cat (supplementary figs. S9 and S10C, Supplementary Material online).

In addition to dissecting tigrina relationships, our analyses also helped understand the effects of including hybrid individuals in SNP-based phylogenetic inference (supplementary fig. S6, Supplementary Material online). In the case of the pampas cat versus S tigrina hybrid (LCO.2), its inclusion rendered the pampas cat paraphyletic, as it was drawn with high support towards the focal clade comprising the tigrina complex, Geoffroy’s cat, and huiña. The other individual detected as a hybrid with our SNP data (bLti135) also led to distortions in the topology, as it was drawn to the Geoffroy’s cat clade, disrupting S tigrina monophyly, altering the position of the huiña, and lowering support for the affected nodes. Inclusion of another putative hybrid (bLge094) between Geoffroy’s cat and S tigrina (previously inferred with traditional markers, but not with this SNP data set) also led to a distortion in the huiña’s position. In this case, the distortion may also have been induced by the extensive amount of missing data for bLge094. The presence of missing data also seems to affect the position of the Andean mountain cat, as previously noted by Li et al. (2016) when using these same SNP data (relative to a larger data set employed in that study). Regardless of its local instability, the Andean mountain cat was strongly supported as being more closely related to the ocelot and margay than to our focal clade comprising the tigrina complex, Geoffroy’s cat, and huiña (supplementary figs. S6–S9, Supplementary Material online), so that its local instability does not affect our conclusions.

Our final phylogenetic analyses (excluding putative hybrids and individuals with extensive missing data) provided robust support for all nodes pertaining to the focal clade (fig. 1*B*; see Supplementary Information, Supplementary Material online for additional analyses). Molecular dating analyses indicated that genus *Leopardus* began its diversification *ca.* 4.6 MYA, and that the pampas cat diverged from the focal clade >4 MYA (see fig. 1*B*). The Central American tigrina diverged from the inner group *ca.* 2.4 MYA, and clearly represents a distinct, species-level lineage. Within the inner group, the divergence between NE tigrina and *L. guttulus* was estimated at *ca.* 1.5 MYA, similar to the depth between the huiña and Geoffroy’s cat (*ca.* 1.8 MYA). This result adds weight to the recognition of these two tigrina units as distinct species, corroborating previous genetic data indicating lack of gene flow between them (Trigo et al. 2013) and morphological analyses that support this taxonomic separation (Nascimento and Feijó 2017). Therefore, we conclude that the tigrina complex comprises at least three different species, one of which (S tigrina) has already been formally recognized as *L. guttulus*. The taxonomic assignment of NE tigrinas and Central American tigrinas will depend on additional geographic sampling of the complex, especially in the Guiana shield, which includes the type locality for *L. tigrinus* (Kitchener et al. 2017; Nascimento and Feijó 2017). This region remains unsampled for molecular data, and holds the key for resolving the taxonomy of this complex. Moreover, tigrinas from other regions in northern South America (e.g., Colombia, Peru) must also be analyzed to assess their affinities with the groups identified here. Interestingly, our results suggest that the “tigrina” morphology may be plesiomorphic in this felid clade, leading to the existence of cryptic species that have remained undetected for decades. More broadly, our results illustrate how genomic data can be used to dissect complex histories of speciation and hybridization, uncover cryptic diversity, and inform the design of phylogenetic analyses in the face of potentially challenging confounding factors.

## Materials and Methods

Our initial data set comprised all *Leopardus* individuals sampled by Li et al. (2016), which were genotyped with an Illumina array targeting genome-wide SNPs identified in the domestic cat (Mullikin et al. 2010). We complemented this data set by genotyping the same markers in five additional *L. geoffroyi* individuals, two of which had suggestive evidence of admixture with *L. tigrinus* from the Brazilian northeast (Trigo et al. 2013) and six additional *L. guttulus* individuals (a species that had not been included in Li et al.’s [2016] study) with known geographic origin. This represents the most complete data set assembled so far for this genus, including 22 individuals from all currently recognized species (supplementary table S1). Beginning from the 62,771 sites surveyed by this array, we applied filters using PLINK (Purcell et al. 2007), excluding individuals with more than 10% of missing data, and sites with 10% missing genotypes. Although the sites were originally selected in the domestic cat as SNPs, in our *Leopardus* data sets most of them (>90%) were invariant, which fits the goal of randomly surveying genomic sites, while still allowing the recovery of substantial evolutionary information. From the full genotype matrix, we constructed several different data sets that varied in the inclusion of putative hybrids and in the treatment of heterozygous and invariant sites (see Supplementary Information, Supplementary Material online for details).

To characterize the genetic structure, we conducted a principal component analysis (PCA) using SmartPCA within the EIGEINSOFT package (Patterson et al. 2006). PCA plots (for PCs 1–10) were then generated in R. Unsupervised analyses with Admixture (Alexander et al. 2009) were performed with three taxon subgroups: subgroup 1 included the focal clade (tigrina complex, Geoffroy’s cat, and huiña) and the pampas cat; subgroup 2 included the tigrina complex and Geoffroy’s cat; and subgroup 3 included S tigrina, NE tigrina, Geoffroy’s cat, and huiña. To reduce bias, we implemented the penalized estimation using the best-fit lambda for each subgroup. All Admixture analyses were performed with a 5-fold cross-validation; the cross-validation error was calculated to determine the best-fitting K value.

For the phylogenetic analyses, we used two different approaches: 1) a concatenation of all sites (both variable and invariant) into a single supermatrix, followed by ML analyses with both RAxML v.8.2.5 (Stamatakis 2006) and IQ-TREE V2.1.2 (Nguyen et al. 2015); and 2) two coalescent-based methods that allow SNP sites to evolve independently: the Bayesian approach implemented in SNAPP (Bryant et al. 2012), and the quartet-based inference implemented in SVDquartets v4.0 (Chifman and Kubatko 2014). Finally, we used mcmctree, included in the PAML 4.9 package (Yang 2007), to date the inferred divergences, using a correlated rates model and a conservative molecular calibration for the root node (base of *Leopardus*), which was derived from the lower and upper boundaries (1.64 MYA and 5.03 MYA, respectively) reported by Li et al. (2016) for the age of this split.

## Supplementary Material

Supplementary data are available at *Molecular Biology and Evolution* online.

## Supplementary Material

msab222_Supplementary_Data
