# Supplementary material for: Genome-Wide SNPs Clarify a Complex Radiation and Support Recognition of an Additional Cat Species
Source: Mol Biol Evol. 2021 Jul 28;38(11):4987–91. doi: 10.1093/molbev/msab222 (PMC8557425; doi:10.1093/molbev/msab222)
Supplement: msab222_Supplementary_Data [file msab222_supplementary_data.docx]

**Supplementary Information for “Genome-wide SNPs clarify a complex radiation and support recognition of an additional cat species”**

Fernanda J. Trindade, Maíra R. Rodrigues, Henrique V. Figueiró, Gang Li, William Murphy, Eduardo Eizirik

**Supplementary Text: Materials, Methods and Results**

The dataset used in this study comprised all *Leopardus* individuals (11) reported by Li et al. (2016), in addition to one *Leptailurus serval* individual (also reported in that study) to be used as outgroup. We further added 11 new individuals of *Leopardus guttulus* and *L. geoffroyi* genotyped specifically for this study (Table S1, Figure S1) using the same laboratory procedures reported by Li et al. (2016).

The 23 individuals were genotyped for genome-wide markers using an Illumina array developed to target 62,771 domestic cat SNPs (Mullikin et al. 2010). An earlier study has demonstrated that this array can be used to survey informative genome-wide variation across all Felidae species (Li et al. 2016), although many of the domestic cat SNPs were found not to be variable in other felids. Therefore, although these sites were identified as SNPs in the domestic cat, most of them are not polymorphic in the other cat species, and are in fact invariant even across species belonging to other felid clades. This is the case in *Leopardus*, a clade in which less than 10% of these sites are variable (see below for the number of variable sites in our different datasets).

Beginning from this dataset, we applied filters using PLINK (Purcell et al. 2007), excluding individuals with more than 10% of missing data, and sites with 10% missing genotypes. In this sense, for the populational analyses mentioned below (and in the Materials and Methods section) we removed bLge094 and OJA.1, as well PSE.12 since it is external to our target genus. The final dataset for the populational analyses contained 3,335 variable sites and 20 individuals (see Table S1 for the list of individuals used in each analysis). To characterize genetic structure, we conducted a Principal Component Analysis (PCA) using SmartPCA within the EIGEINSOFT package (Patterson et al. 2006). PCA plots (for PCs 1-10) were then generated in R. The most valuable observation that we made with the results was the clear separation of the tigrina complex into three distinct clusters (Figure S2).

Unsupervised analyses with Admixture (Alexander et al. 2009) were performed with three taxon subgroups: subgroup 1 included the focal clade (tigrina complex, Geoffroy’s cat and huiña) and the pampas cat; subgroup 2 included the tigrina complex and Geoffroy’s cat; and subgroup 3 included S tigrina, NE tigrina, Geoffroy’s cat and huiña. The aim of splitting the individuals into these subgroups was to test if the ancestry distinction among the tigrina units would remain robust independently of the set of surveyed species. To reduce bias in the Admixture analyses, we implemented the penalized estimation using the best-fit lambda for each subgroup. All Admixture analyses were performed with a 5-fold cross-validation; the cross-validation error was calculated to determine the best-fitting K value. As mentioned above for the PCA results, considering the best-fitting K value for all analyses, the tigrina complex showed three distinct ancestry profiles (Figures S3-S5).

For the phylogenetic analyses, we iteratively tested the exclusion of hybrid individuals, the coding of heterozygous sites as ambiguities or as missing data, and the use of invariant sites. Since our full dataset contained hybrid individuals between *L. guttulus* and *L. geoffroyi*, as well as between *L. guttulus* and *L. colocola* (Table S1), to assess how much they could affect our phylogenetic inferences, we performed several ML analyses including one hybrid at time and all of them together (Figure S6). Based on these results, we decided to remove from the final analyses the captive hybrid and the hybrids whose inclusion decreased nodal support, especially for our target group (the tigrina complex and its immediate relatives). In addition, we explored different approaches to incorporate heterozygous sites contained in our data matrix: (i) using only one of alleles sampled in each individual; (iii) sampling the other allele per individual; and (iii) integrating the allelic information for each individual, i.e. replacing the heterozygous sites by standard ambiguity codes (Figure S7). For each of these tests, we employed RAxML v.8.2.5 (Stamatakis 2006) with 1000 bootstrap replicates, using the GTRGAMMA model, which was estimated as the best-fit model with JModelTest2 (Darriba et al. 2012). This masking approach had been used before by Li et al. (2016), with an overlapping dataset, as well by other studies (e.g. Chaplin et al., 2019; Dupuis et al., 2017), and yielded the topology with the highest nodal support for the target group in this study. Therefore, our main phylogenetic dataset included both variable and invariant sites, with heterozygous sites coded as ambiguous, and excluded sites with >10 individuals exhibiting missing data.

In the process of assessing the impact of including hybrids in the analyses, as well as different coding schemes for heterozygous sites, we explored several different datasets. These encompassed datasets that included each putative hybrid separately, as well as all jointly (see Figure S6); and datasets with different options of including heterozygous sites or coding them as ambiguous, as well including or not the *L. serval* outgroup, with both a supermatrix (Figures S7 and S8) and a Bayesian coalescent (Figure S9) approach. Throughout these analyses, we observed that the topology was very robust, especially with respect to the paraphyly of the tigrina complex.

Furthermore, considering the potential issues of performing phylogenetic analyses with SNP-based datasets (Leaché et al., 2015), we additionally explored other approaches to infer *Leopardus* relationships with our data. In addition to the main RAxML analyses, which used both variable and invariant sites with the best-fit model estimated with jModelTest2 (Figures 1B, S6, S7, S8), we also filtered out all invariant sites and performed an acquisition bias correction in RAxML, using the conditional likelihood approach (Leaché et al., 2015)(Figure S10A). In addition, we analyzed the full dataset with IQ-TREE v2.1.2 (Nguyen et al., 2015), using the best-fit model estimated with ModelFinder (Kalyaanamoorthy et al., 2017) (Figure S10B), as well coalescent-based reconstructions using the software SNAPP (Bryant et al. 2012), implemented in BEAST2, and SVDquartets v4.0 (Chifman and Kubatko, 2014), implemented in PAUP*. The model used in IQ-TREE was K3Pu+F+R2 and we performed 1,000 rapid bootstrap replicates. For the SVDquartets analysis, the monophyly of each species was first checked and then species collapsed into a single terminal branch each (Figure S10C).

Finally, we used mcmctree, included in the PAML 4.9 package (Yang 2007), to date the inferred divergences using the topology inferred by RAxML, using a correlated rates model and a conservative molecular calibration for the root node (base of *Leopardus*), which was derived from the lower and upper boundaries (1.64 MYA and 5.03 MYA, respectively) reported by Li et al. (2016) for the age of this split.

Supplementary Table S1. Information on the samples included in this study. The underlined individuals had been previously identified as potential hybrids based on traditional molecular markers (Trigo et al. 2008, 2013). The asterisk indicates individuals that were not included in the genetic clustering analysis due to extensive missing data (or for being the outgroup). The ‘^†^’ indicates individuals that were removed from the final Maximum Likelihood phylogenetic reconstruction (Figure 1B) according to Figure S6 results. The ‘^α^’ indicates individuals comprising the focal group for PCA analysis (Supplementary Figure S2). The ‘^β^’ indicates subgroup 2 individuals for the Admixture analysis (Supplementary Figure S4). The ‘^δ^’ indicates subgroup 3 individuals for the Admixture analysis (Supplementary Figure S5). Within the present dataset, we define the “tigrina complex” as comprising the individuals of Southern tigrina (*L. guttulus*), NE tigrina (*L. tigrinus*) and Central American tigrina (*L. tigrinus*). ZSS: Zoo Sapucaia do Sul, Brazil; LBGM: Laboratório de Biologia Genômica e Molecular, PUCRS; FZB-RS: Fundação Zoobotânica do Rio Grande do Sul, Brazil.

| **Species** | **Latin name** | **Individual ID** | **Geographic origin** | **Sex** | **Year** | **Sample source** | **Data source** |
| --- | --- | --- | --- | --- | --- | --- | --- |
| Geoffroy’s cat | *Leopardus geoffroyi* | bLge031^α,β,δ^ | Quaraí, RS/Brazil | - | 1998 | ZSS | This study |
|  |  | bLge036^α,β,δ^ | Rio Grande, RS/Brazil | F | - | LBGM | This study |
|  |  | bLge075^α,β,δ^ | Arroio Grande, RS/Brazil | F | 2003 | LBGM | This study |
|  |  | bLge077^α,β,δ^ | Dom Pedrito, RS/Brazil | M | 2004 | LBGM | This study |
|  |  | bLge094*^,†^ | Rio Grande do Sul – Brazil | F | - | FZB-RS | This study |
|  |  | OGE.3^α,β,δ^ | - | - | - | - | Li et al., 2016 |
|  |  | OGE.1^α,β,δ^ | - | - | - | - | Li et al., 2016 |
| Southern tigrina | *L. guttulus* | bLti102^α,β,δ^ | RS/Brazil | F | 2004 | ZSS | This study |
|  |  | bLti124^α,β,δ^ | Arroio do Meio, RS/Brazil | M | 2003 | LBGM | This study |
|  |  | bLti135^†,α,β,δ^ | Estância Velha, RS/Brazil | F | 2004 | ZSS | This study |
|  |  | bLti138^α,β,δ^ | Machadinho, RS/Brazil | F | 2000 | FZB-RS | This study |
|  |  | bLti140^α,β,δ^ | Ibarama, RS/Brazil | M | 2006 | FZB-RS | This study |
|  |  | bLti160^α,β,δ^ | Urubici, SC/Brazil | M | 2006 | LBGM | This study |
| NE tigrina | *L. tigrinus* | LTI.6^α,β,δ^ | NE Brazil | - | - | - | Li et al., 2016 |
|  |  | LTI.1^α,β,δ^ | NE Brazil | - | - | - | Li et al., 2016 |
| Central American tigrina | *L. tigrinus* | LTI.13^α,β^ | Costa Rica | - | - | - | Li et al., 2016 |
| Pampas cat | *L. colocola* | LCO.2^†^ | - | - | - | - | Li et al., 2016 |
|  |  | LCO.3 | - | - | - | - | Li et al., 2016 |
| Andean mountain cat | *L. jacobita* | OJA.1*^,†^ | - | - | - | - | Li et al., 2016 |
| Ocelot | *L. pardalis* | LPA.6 | - | - | - | - | Li et al., 2016 |
| Margay | *L. wiedii* | LWI.22 | - | - | - | - | Li et al., 2016 |
| Huiña | *L. guigna* | OGU.3^α,δ^ | - | - | - | - | Li et al., 2016 |
| Serval | *Leptailurus serval* | PSE.12*^,†^ | - | - | - | - | Li et al., 2016 |


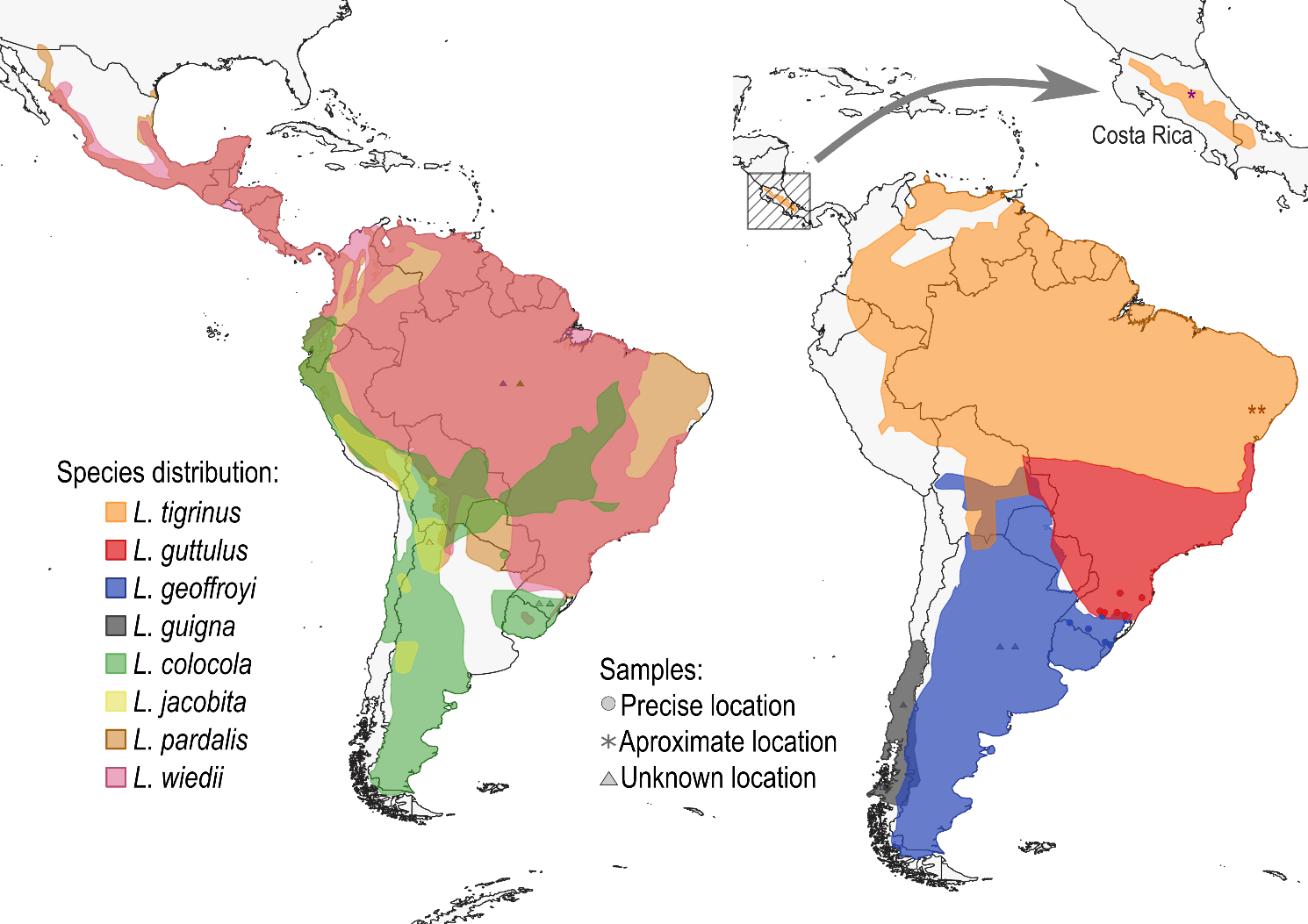


Supplementary Figure S1. *Leopardus* species distribution maps according to the IUCN database; sampling locations (according to Supplementary Table S1) are indicated. The circles and asterisks mark the precise or approximate sample locations, respectively; triangles represent samples from zoo animals with unknown provenance, whose location was placed at a central position within the putative regional source of the individuals. The tigrina complex includes *L. tigrinus* and *L. guttulus* distributions. It is noteworthy that the current *L. tigrinus* distribution seems to harbor at least two different tigrina species, based on the results from this study, along with previous genetic, morphological and ecological data (see text for details). Likewise, the *L. colocola* range depicted here includes the distribution of five recently proposed distinct species (Nascimento et al. 2021).


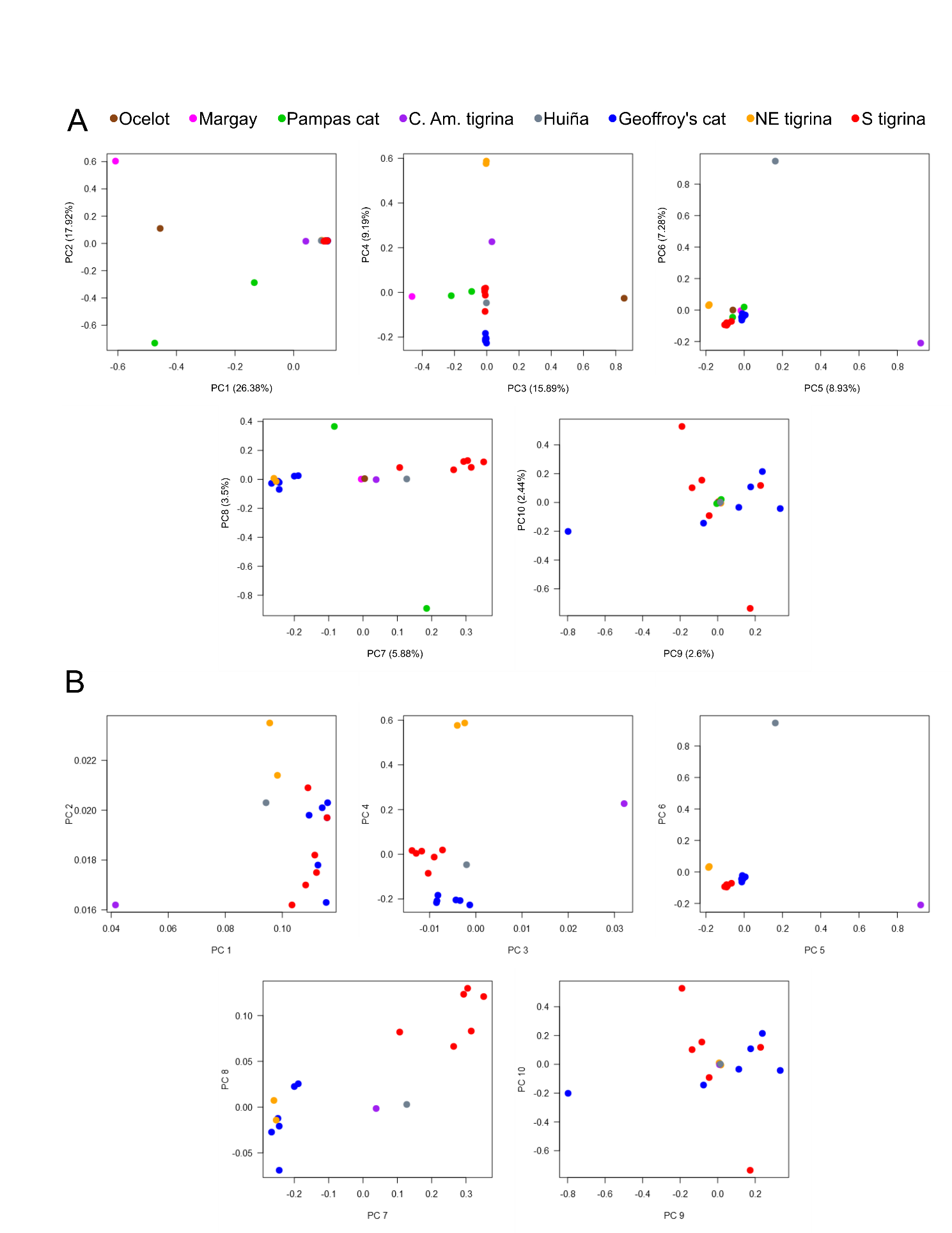


Supplementary Figure S2. Principal Component Analysis (PCA) results for genome-wide SNP data for genus *Leopardus*. A) Results for PCs 1-10 for a broader taxonomic sample encompassing eight evolutionary units (species or potential species); B) Results for PCs 1-10 for the focal taxonomic group encompassing the tigrina complex, Geoffroy’s cat and the huiña.


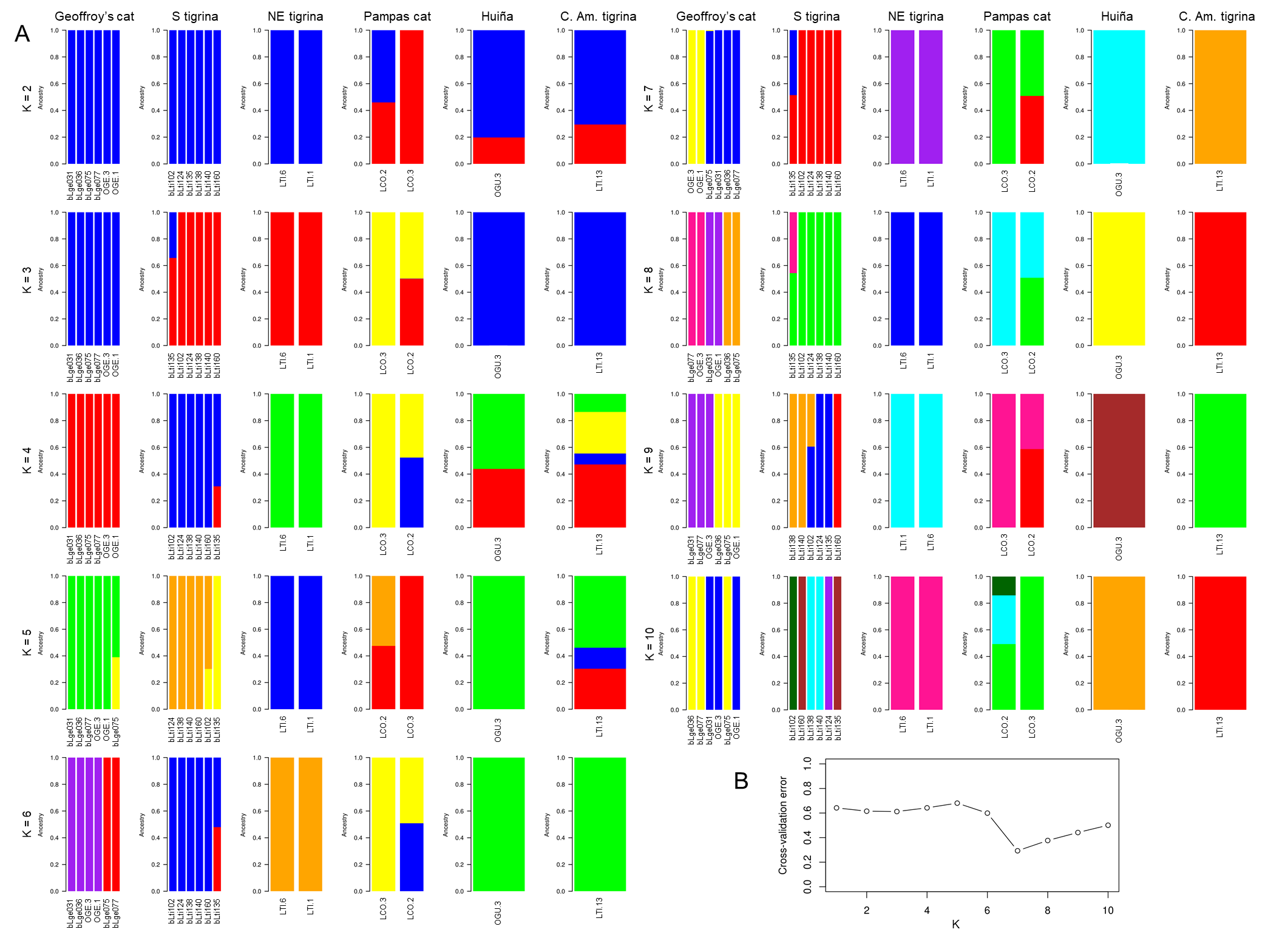


Supplementary Figure S3. A) Admixture results for taxon subgroup 1, encompassing six evolutionary units (species or potential species), identified at the top. Each plot assumes a different number of distinct genetic clusters (K), identified on its left side. B) Plot of the cross-validation error for this analysis. Here we used penalized lambda 8, the best-fitting lambda for this taxon set (estimated using K = 6, the number of potential units). According to the associated cross-validation error (B), the best K would be 7.


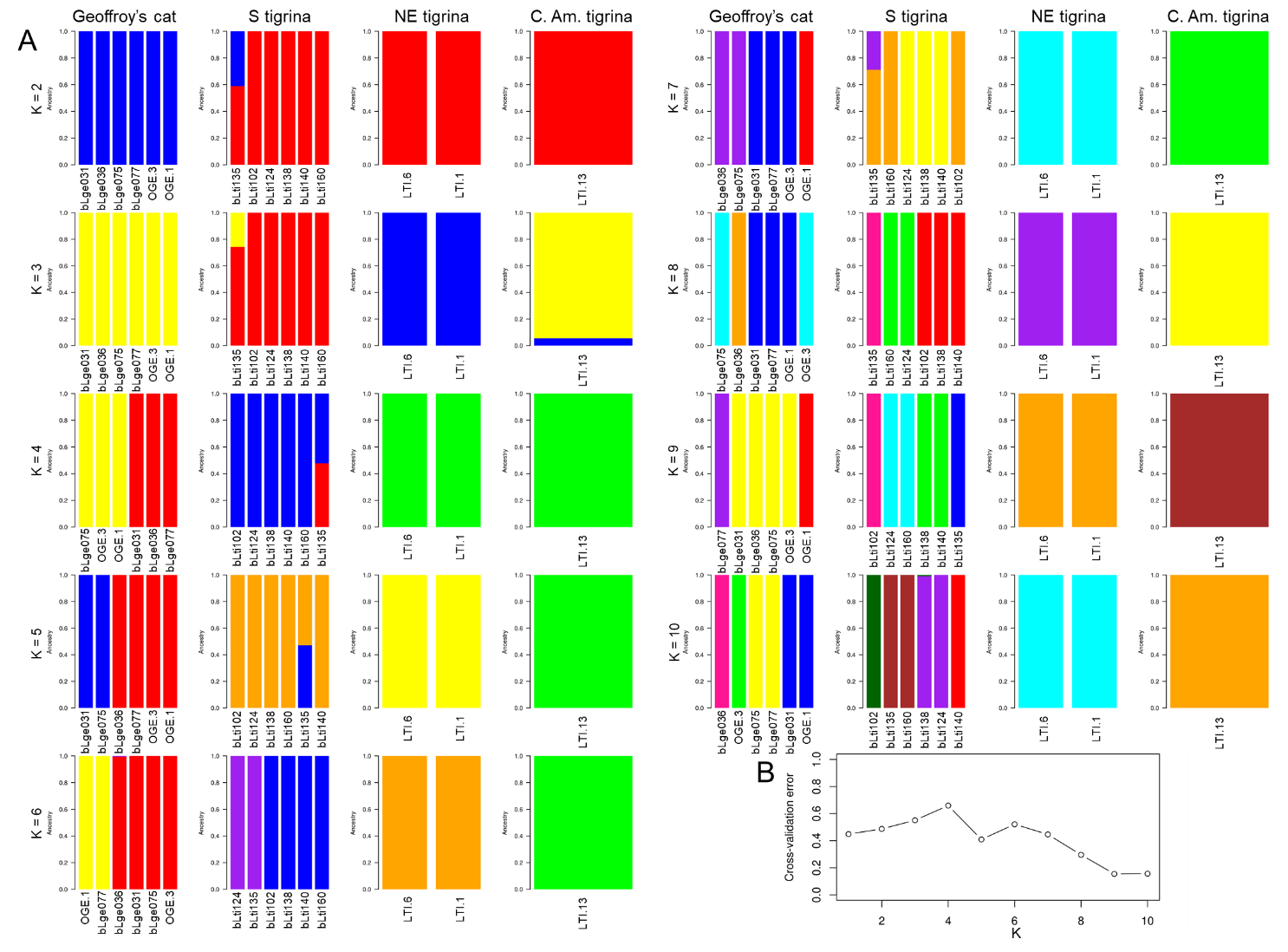


Supplementary Figure S4. A) Admixture results for subgroup 2, encompassing the tigrina complex and Geoffroy’s cat (evolutionary units are identified at the top). Each plot assumes a different number of distinct genetic clusters (K), identified on its left side. B) Plot of the cross-validation error for this analysis. Here we used penalized lambda 8, the best-fitting lambda for this taxon set (estimated using K = 4, the number of potential units). According to the associated cross-validation error (B), the best K would be 9, although K=5 is more interpretable biologically.


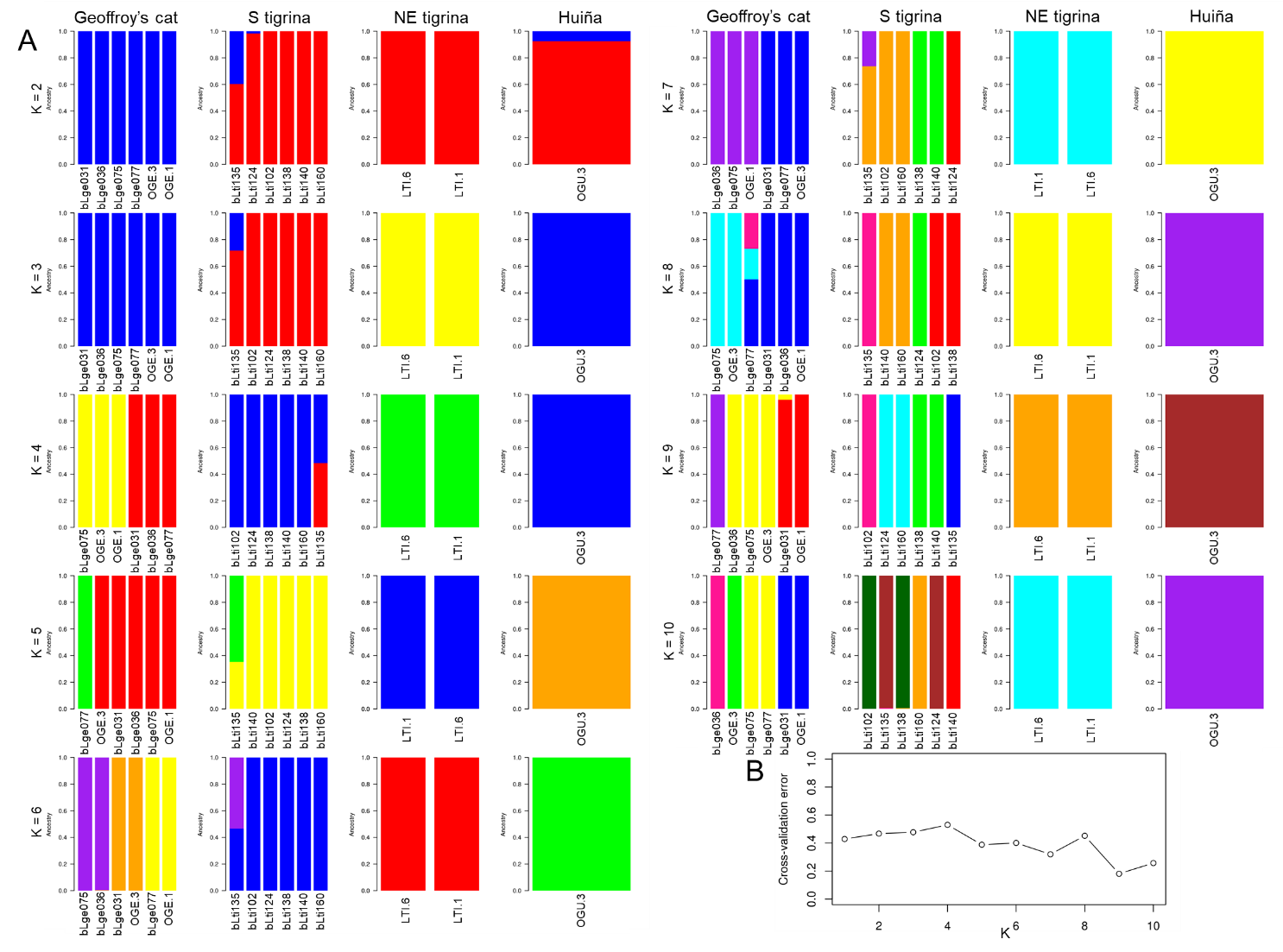


Supplementary Figure S5. A) Admixture results for subgroup 3, encompassing two South American units within the tigrina complex (S tigrina and NE tigrina), along with Geoffroy’s cat and the huiña (evolutionary units are identified at the top). Each plot assumes a different number of distinct genetic clusters (K), identified on its left side. B) Plot of the cross-validation error for this analysis. Here we used penalized lambda 4, the best-fitting lambda for this taxon set (estimated using K = 4, the number of potential units). According to the associated cross-validation error (B), the best K would be 9, although K=5 is more interpretable biologically.


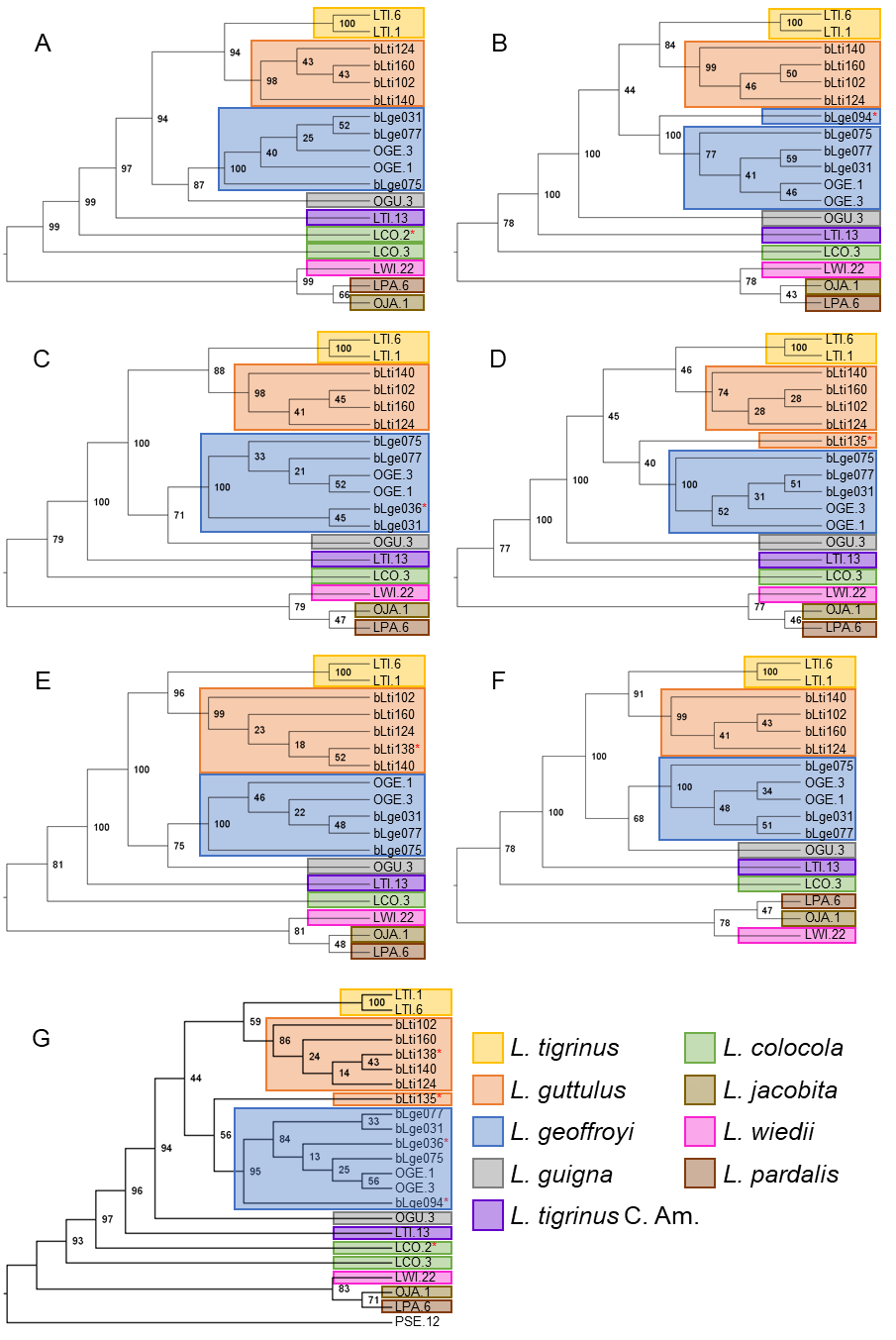


Supplementary Figure S6. Maximum likelihood (ML) phylogenies based on a supermatrix of concatenated genome-wide nucleotide sites, with different sets of *Leopardus* individuals including inferred inter-species hybrids (identified with a red asterisk in each tree). In these analyses, heterozygous sites were masked as missing data; up to 10 terminal taxa with missing data per site were allowed, resulting in a dataset comprising 58,477 sites. Numbers next to nodes are bootstrap support values for the respective clade. The species are identified by box colors; individual IDs follow Table S1. The taxon sets were as follows: (A) Inclusion of LCO.2, a captive-bred F1 hybrid between the pampas cat and *L. guttulus* (see main text and Supplementary Figure S3); (B-E) Inclusion of free-ranging individuals from southern Brazil that were inferred to be hybrids between Geoffroy’s cat and S tigrina based on traditional molecular markers (Trigo et al., 2013); only individual bLti135 (shown in D) was identified as a hybrid based on our genome-wide markers; (F) no putative hybrids included; (G) all putative hybrids included, as well as the outgroup *Leptailurus serval* (PSE.12). Of the total matrix, there were 2986 variable sites in A, 3170 variable sites in B, 2956 variable sites in C, 3052 variable sites in D, 2957 variable sites in E, 2814 variable sites in F, and 4274 variable sites in G.


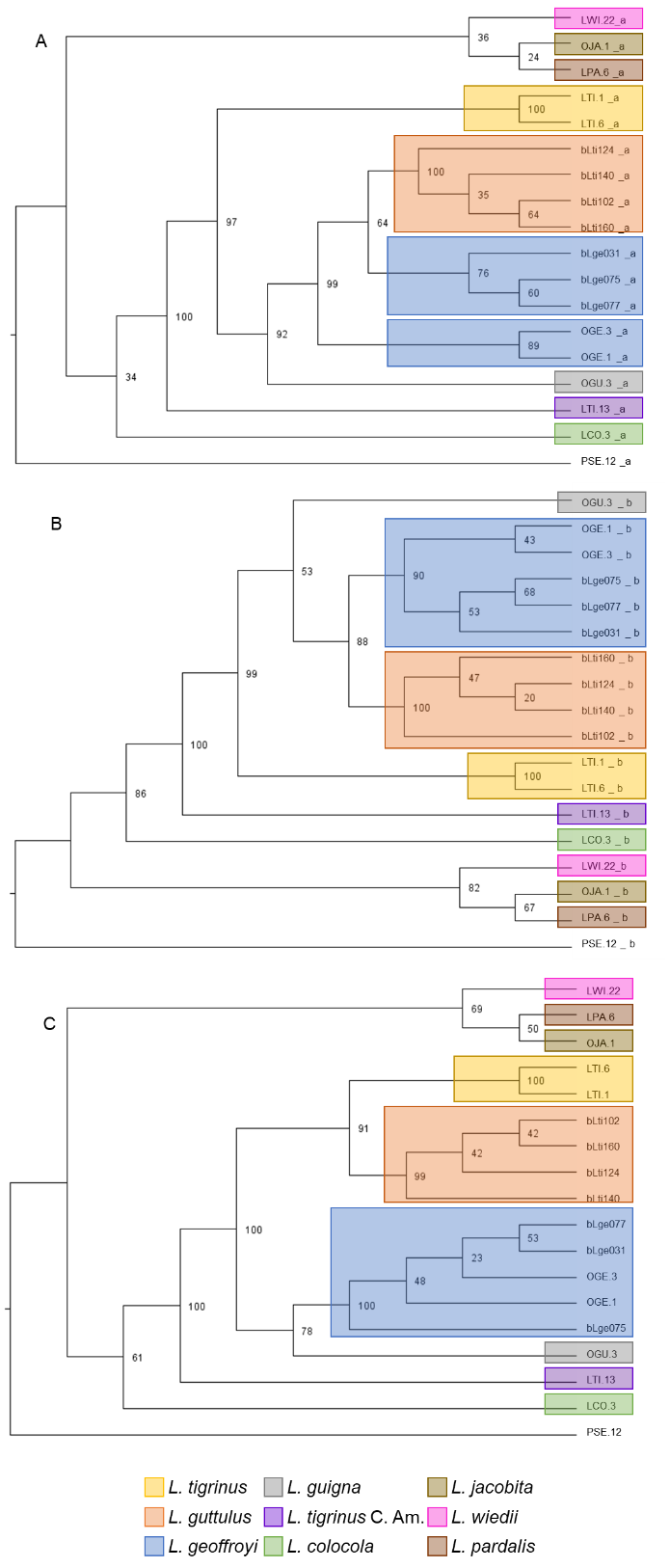


Supplementary Figure S7. Maximum likelihood (ML) phylogenies based on a supermatrix of 61,955 concatenated genome-wide nucleotide sites, with different allelic combinations for the heterozygous sites. The allelic combinations were as follows: (A-B) Only one (A) or the other (B) allele at each site was sampled, respectively; (C) Heterozygous sites were coded as ambiguous. In these analyses all species were included, without any putative hybrids, and *Leptailurus serval* (PSE.12) was used as the outgroup. Of the total matrix, there were 5741 variable sites in A, 5068 variable sites in B, and 5740 variable sites in C. Numbers next to nodes are bootstrap support values for the respective clade. *Leopardus* species are identified by box colors; individual IDs follow Table S1.


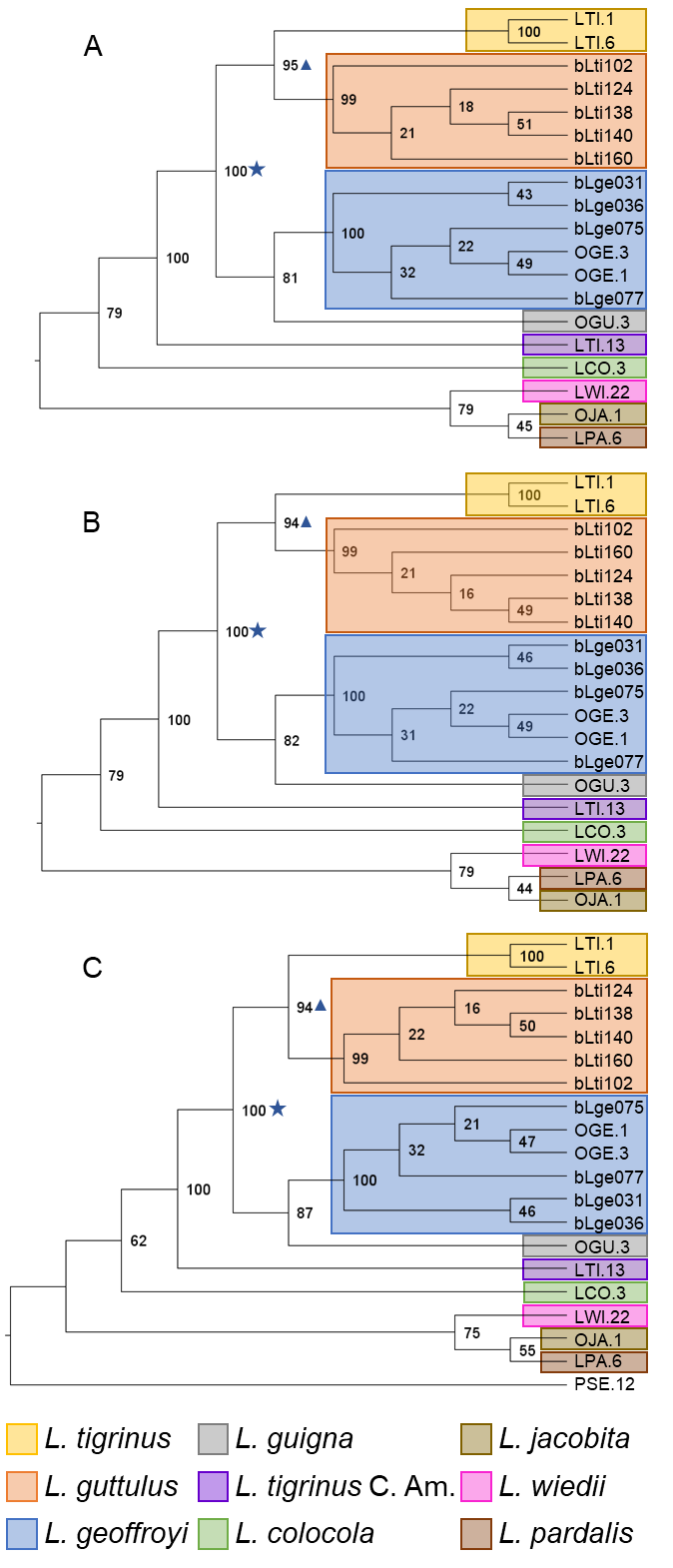


Supplementary Figure S8. Maximum likelihood (ML) phylogenies based on a supermatrix of concatenated genome-wide nucleotide sites, exploring different schemes for masking heterozygous sites and the inclusion of an outgroup. Heterozygous sites were masked as missing (A) or ambiguous (B). In (C) they were masked as ambiguous, and the serval (*Leptailurus serval*, PSE) was included as an outgroup. Up to 10 terminal taxa with missing data per site were allowed, resulting in datasets comprising 58,477 sites in (A) and 58,905 sites in (B) and (C). Of the total matrices, there were 3084 variable sites in A, 3945 variable sites in B, and 4477 variable sites in C. Numbers next to nodes are bootstrap support values for the respective clade. *Leopardus* species are identified by box colors; individual IDs follow Table S1. Individuals with extensive missing data and/or identified as hybrids with our SNP dataset (see Supplementary Table S1 and Supplementary Figure S3-S6) were excluded, except for the Andean mountain cat, which was kept to assess its phylogenetic stability with these datasets. The node defining S tigrina and NE tigrina as sister-species is marked by a triangle; the node supporting paraphyly of the tigrina complex due to the external position of the Central American tigrina is indicated by a star.


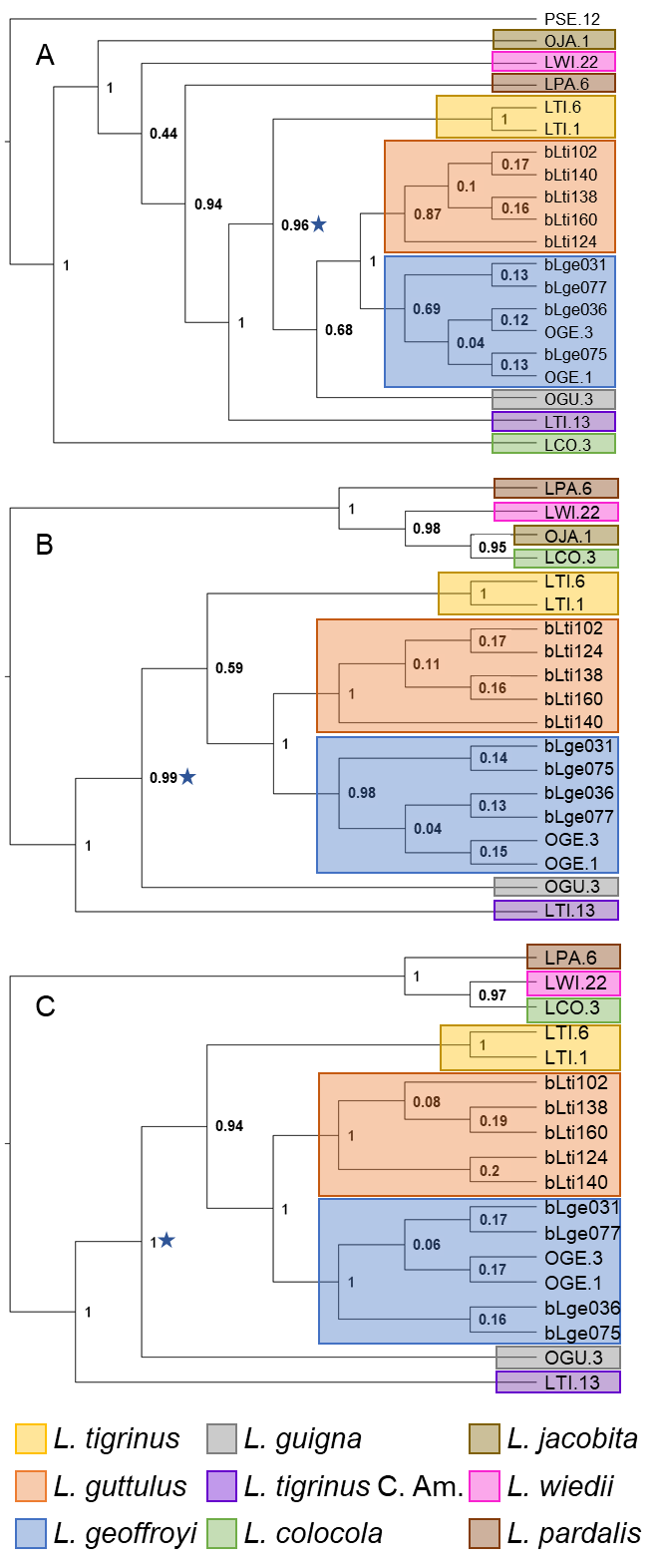


Supplementary Figure S9. Coalescent species trees reconstructed with the Bayesian approach implemented in SNAPP (see Materials and Methods), which considers each SNP site as independently evolving. Heterozygous sites were excluded in (A) and (B), and the serval was included as an outgroup in (A). In (C) heterozygous sites were coded as ambiguous and the Andean mountain cat was excluded due to its extensive missing data. Up to ~2% of missing data per individual were allowed; monomorphic and missing sites were excluded. These coding and filtering options yielded 2035 SNPs in A, 1150 SNPs in B, and 1282 SNPs in C. *Leopardus* species are identified by box colors; individual IDs follow Table S1. In these analyses, NE tigrina and S tigrina were not reconstructed as sister-species. The node further supporting paraphyly of the tigrina complex (due to the external position of the Central American tigrina) is indicated by a star, as in Supplementary Figure S8.


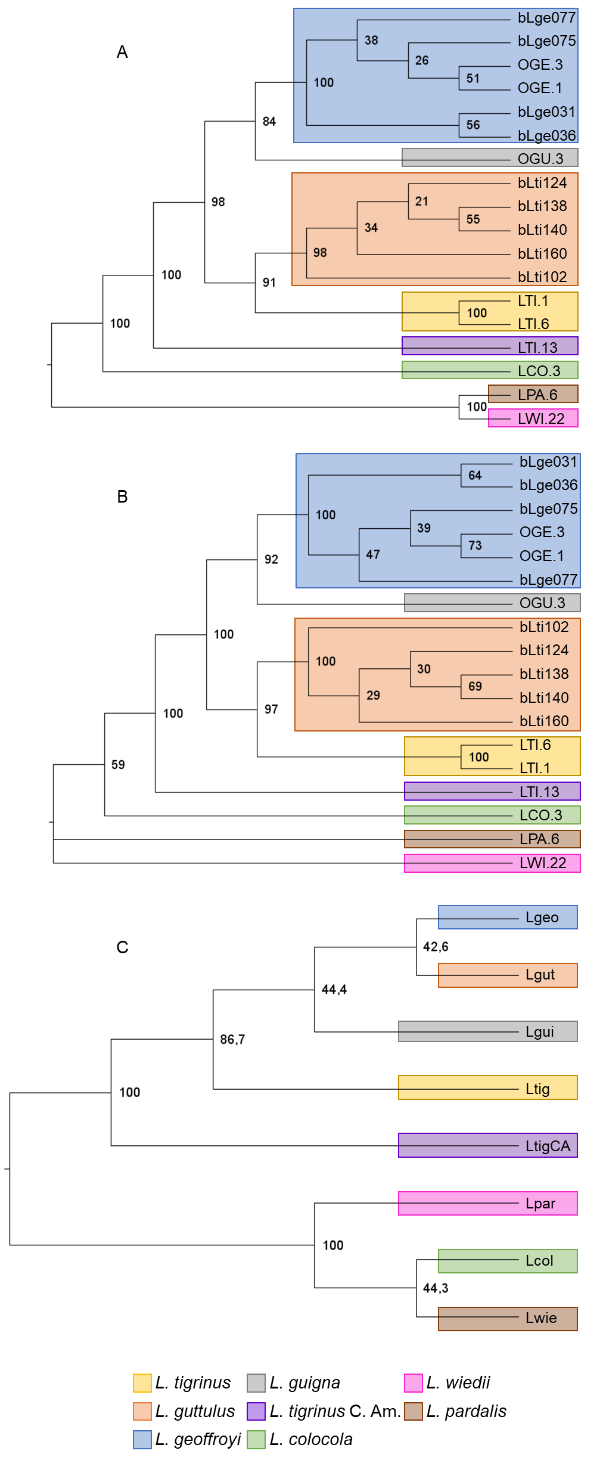


Supplementary Figure S10. Phylogenetic reconstructions using complementary supermatrix and coalescent-based approaches. The set of individuals and sequences used here were the same for Figure 1B, except that in (A) there only the variable sites from that dataset were included. (A) Maximum likelihood phylogeny using RAxML, based on a supermatrix of concatenated variable genome-wide SNPs (1982 sites), applying an acquisition bias correction model using the conditional likelihood option; the node numbers are bootstrap support values. (B) Maximum likelihood phylogeny using IQ-TREE, based on a supermatrix of concatenated genome-wide sites, with the substitution model inferred by ModelFinder; node numbers are bootstrap support values. (C) Coalescent species tree reconstructed with SVDquartets; node numbers are bootstrap support values; individuals from the same species were collapsed. *Leopardus* species are identified by box colors; individual IDs follow Table S1.


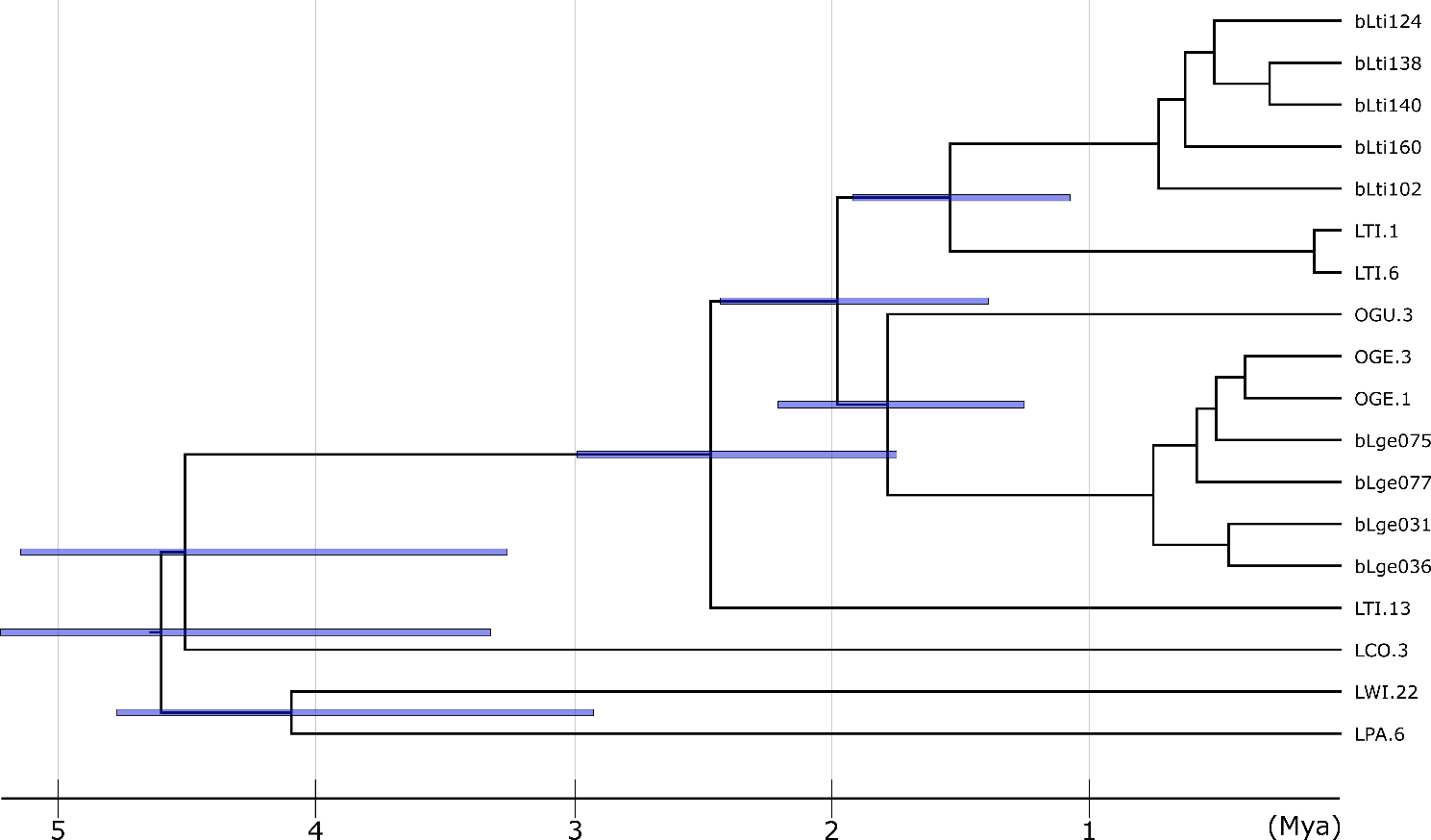


Supplementary Figure S11. Phylogeny presented in Figure 1B, with true node heights across the whole tree (i.e. not cropping a long branch). Node bars represent time intervals in million years ago (Mya).

References:

Alexander DH, Novembre J, Lange K. 2009. Fast model-based estimation of ancestry in unrelated individuals. Genome Res. 19(9):1655–1664. doi:10.1101/gr.094052.109.

Bryant D, Bouckaert R, Felsenstein J, Rosenberg NA, Roychoudhury A. 2012. Inferring species trees directly from biallelic genetic markers: Bypassing gene trees in a full coalescent analysis. Mol Biol Evol. 29(8):1917–1932. doi:10.1093/molbev/mss086.

Chaplin K, Sumner J, Hipsley CA, Melville J. 2020. An integrative approach using phylogenomics and high-resolution x-ray computed tomography for species delimitation in cryptic taxa. Systematic Biology, 69(2), 294-307.

Darriba D, Taboada GL, Doallo R, Posada D. 2012. JModelTest 2: More models, new heuristics and parallel computing. Nat Methods. 9(8):772. doi:10.1038/nmeth.2109.

Dupuis JR, Brunet BMT, Bird HM, Lumley LM, Fagua G, Boyle B, ... Sperling FAH. 2017. Genome-wide SNPs resolve phylogenetic relationships in the North American spruce budworm (Choristoneura fumiferana) species complex. Molecular Phylogenetics and Evolution, 111, 158-168.

IUCN 2021. The IUCN Red List of Threatened Species. Version 2021-1. https://www.iucnredlist.org. Downloaded on [June 2021].

Kalyaanamoorthy S, Minh BQ, Wong TK, Von Haeseler A, Jermiin LS. 2017. ModelFinder: fast model selection for accurate phylogenetic estimates. Nature methods, 14(6), 587-589. doi: 10.1038/nmeth.4285.

Leaché AD, Banbury BL, Felsenstein J, De Oca, ANM, Stamatakis, A. 2015. Short tree, long tree, right tree, wrong tree: new acquisition bias corrections for inferring SNP phylogenies. Systematic biology, 64(6), 1032-1047. doi: 10.1093/sysbio/syv053.

Li G, Davis BW, Eizirik E, Murphy WJ. 2016. Phylogenomic evidence for ancient hybridization in the genomes of living cats (Felidae). Genome Res. 26(1):1–11. doi:10.1101/gr.186668.114.

Mullikin JC, Hansen NF, Shen L, Ebling H, Donahue WF, Tao W, Saranga DJ, Brand A, Rubenfield MJ, Young AC, et al. 2010. Light whole genome sequence for SNP discovery across domestic cat breeds. BMC Genomics. 11(1):1–8. doi:10.1186/1471-2164-11-406.

Nascimento FO do, Feijó A. 2017. Taxonomic revision of the tigrina Leopardus tigrinus (Schreber, 1775) species group (carnivora, felidae). Pap Avulsos Zool. 57(19):231–264. doi:10.11606/0031-1049.2017.57.19.

Nascimento FO Do, Cheng J, Feijó A. 2021. Taxonomic revision of the pampas cat Leopardus colocola complex (Carnivora: Felidae): an integrative approach. Zoological Journal of the Linnean Society, 191(2), 575-611. doi:10.1093/zoolinnean/zlaa043.

Nguyen LT, Schmidt HA, Von Haeseler A, Minh BQ. 2015. IQ-TREE: a fast and effective stochastic algorithm for estimating maximum-likelihood phylogenies. Molecular biology and evolution, 32(1), 268-274. doi:10.1093/molbev/msu300.

Patterson N, Price AL, Reich D. 2006. Population structure and eigenanalysis. PLoS Genet. 2(12):2074–2093. doi:10.1371/journal.pgen.0020190.

Purcell S, Neale B, Todd-Brown K, Thomas L, Ferreira MAR, Bender D, Maller J, Sklar P, De Bakker PIW, Daly MJ, et al. 2007. PLINK: A tool set for whole-genome association and population-based linkage analyses. Am J Hum Genet. 81(3):559–575. doi:10.1086/519795.

Stamatakis A. 2006. RAxML-VI-HPC: Maximum likelihood-based phylogenetic analyses with thousands of taxa and mixed models. Bioinformatics. 22(21):2688–2690. doi:10.1093/bioinformatics/btl446.

Chifman J, Kubatko L. 2014. Quartet inference from SNP data under the coalescent model. Bioinformatics, 30(23), 3317-3324. doi: 10.1093/bioinformatics/btu530.

Trigo TC, Schneider A, De Oliveira TG, Lehugeur LM, Silveira L, Freitas TRO, Eizirik E. 2013. Molecular data reveal complex hybridization and a cryptic species of Neotropical wild cat. Curr Biol. 23(24):2528–2533. doi:10.1016/j.cub.2013.10.046.

Yang Z. 2007. PAML 4: Phylogenetic analysis by maximum likelihood. Mol Biol Evol. 24(8):1586–1591. doi:10.1093/molbev/msm088.
